# A peptide that binds the Asian citrus psyllid gut reduces transmission of the citrus greening pathogen *Candidatus* Liberibacter asiaticus

**DOI:** 10.1128/aem.00577-26

**Published:** 2026-09-04

**Authors:** Miltan C. Roy, Clebson S. Tavares, Ruchir Mishra, Erik L. Roldan, Lukasz L. Stelinski, Kirsten S. Pelz-Stelinski, Bryony C. Bonning

**Affiliations:** 1 Citrus Research and Education Center, Department of Entomology and Nematology, University of Florida 3463, Lake Alfred, Florida, USA; 2 Department of Entomology and Nematology, University of Florida 3463, Gainesville, Florida, USA; 3 Mid-Florida Research and Education Center, University of Florida 3463, Apopka, Florida, USA; Universite de la Reunion, Ste Clotilde, France

**Keywords:** phage display, gut-binding peptide, transmission, *Candidatus *Liberibacter asiaticus, *Diaphorina citri*

## Abstract

Citrus greening disease, associated with the bacterium *Candidatus* Liberibacter asiaticus (*C*Las), has severely impacted citrus yields on a global scale. *C*Las is vectored by the Asian citrus psyllid, *Diaphorina citri* Kuwayama, in a circulative and propagative manner. Extensive application of chemical insecticides for suppression of psyllid populations has resulted in widespread resistance. Despite considerable effort and expenditure, citrus greening disease continues to impair citrus production. For transmission to occur, *C*Las must bind and infect the psyllid vector via the gut epithelium, but little is known of the specific molecular interactions involved. By screening a phage display library, we selected ten psyllid gut binding peptides, a subset of which have sequence similarity to *C*Las surface proteins. Peptide binding to the surface of the psyllid gut epithelium was confirmed by identification of gut proteins bound by specific peptides. We evaluated whether these peptides could interfere with the interaction between *C*Las and the gut epithelium of *D. citri*, thereby impeding pathogen transmission. Peptide DcNy2 significantly reduced *C*Las acquisition and inoculation by *D. citri* compared to control treatments. Nymphs fed specific peptides exhibited reduced *C*Las acquisition and infection of both the alimentary canal and salivary glands without negatively impacting psyllid fecundity, development, or survival. These results support peptide-mediated disruption of *C*Las transmission by *D. citri* as a potential component of integrated strategies to manage citrus greening disease.

**IMPORTANCE:**

Citrus greening disease, or huanglongbing, causes billions of dollars in losses and represents one of the greatest challenges facing global citrus production. Despite significant investment on multiple fronts, effective disease management strategies are lacking. This study shows for the first time that short peptides that bind to the surface of the gut of the Asian citrus psyllid vector interfere with *C*Las association with the gut. This critical first gut binding step is required for *C*Las to infect and propagate within the psyllid vector. The downstream consequence of this peptide-mediated interference is reduced acquisition and transmission of the citrus pathogen. This work provides the foundation for new transgenic or paratransgenic tools for use in the management of citrus greening disease.

## INTRODUCTION

Citrus greening, or huanglongbing, is a devastating disease threatening global citrus production (1, 2). The disease is associated with three phloem-limited bacteria: *Candidatus* Liberibacter asiaticus (*C*Las), *Ca*. L. americanus (*C*Lam), and *Ca*. L. africanus (*C*Laf), with *C*Las being the most widespread and destructive (1, 3–5). *C*Las is transmitted in a persistent, propagative manner by the Asian citrus psyllid (*Diaphorina citri* Kuwayama) (6, 7), with nymphs more efficient than adults at acquiring and transmitting the pathogen (7–9). Severe reductions in fruit yield and quality and substantial economic losses in major citrus-growing regions have resulted from this disease (2, 3).

Current citrus greening management emphasizes minimizing pathogen transmission by suppressing *D. citri* populations with insecticides, which can provide short-term benefits (10–12). Broad-spectrum antibiotics have also shown some efficacy in reducing *C*Las abundance in vectors (13). However, these approaches are unsustainable in regions with high infection rates, where citrus greening-affected trees do not generate sufficient yield to offset production costs (14). Moreover, reliance on chemical control has led to insecticide resistance and negative environmental consequences (15, 16). In the absence of resistant citrus cultivars, innovative and sustainable strategies for disease management are urgently needed.

*C*Las acquisition begins when psyllids ingest infected phloem sap (7). The bacterium then binds the surface of the gut epithelium and enters gut cells (6), likely via clathrin-independent endocytosis (17). *C*Las accumulates within endoplasmic reticulum-associated vacuoles in the midgut cells (18), the putative site for *C*Las replication (19), and forms biofilms on the hemolymph side of the midgut epithelium (20). The highest bacterial loads within *D. citri* are typically found in the midgut and salivary glands (19, 21). The midgut, therefore, represents a critical barrier for *C*Las invasion (22). Targeting the interaction between *C*Las and *D. citri*, particularly during initial gut binding, represents a promising approach to managing citrus greening by limiting bacterial acquisition and subsequent transmission (3, 23).

Vector-borne pathogens depend on specific molecular interactions with their insect hosts to facilitate acquisition, retention, and transmission (24). Disrupting these interactions using peptides, lectins, or recombinant proteins has shown promise across a range of pathosystems (25–27). Gut-binding peptides (GBPs), identified by screening of phage display libraries (28), can impede pathogen attachment to vector tissues (22). For example, the SM1 peptide blocks *Plasmodium berghei* invasion of the mosquito midgut and salivary glands (29, 30); the peptide GBP3.1 inhibits acquisition of pea enation mosaic virus (PEMV) by the pea aphid (27); and the peptide BBP2.1 reduces entry of deformed wing virus into the honey bee gut (31). These studies underscore the importance of the midgut epithelium as a key point of intervention for blocking pathogen establishment within—and transmission by—vector populations.

For this study, we evaluated whether *D. citri*-specific GBPs can compete with *C*Las for binding sites on the gut epithelium and whether these peptides could be used to disrupt pathogen acquisition and transmission. We evaluated the efficacy of selected GBPs to block *C*Las acquisition and inoculation, while also assessing their impact on vector fitness, including fecundity, development, and survival. Peptides that compete with *C*Las for binding within the *D. citri* gut have potential utility as tools to reduce *D. citri* vector competence, with transgenic or paratransgenic delivery systems envisioned for management of citrus greening disease.

## RESULTS

### Selection of *D. citri* gut-binding peptides

*D. citri* gut-binding peptides were identified as follows: Adult (28) or nymph *D. citri* were fed on a library of phages that display peptides with random amino acids on their surface. Following feeding on the phage display library (Ph.D.-C7C library; New England BioLabs), guts were dissected, unbound phage washed away, and bound phage eluted. Eluted phage were then amplified and fed again to *D. citri* for a second round of enrichment to enrich for phage displaying peptides that bind to the gut surface. All phage eluted from the gut after rounds 1 and 2 of enrichment were sequenced, resulting in thousands of peptide sequences. A custom bioinformatics pipeline (28) was used to identify enriched peptides and remove false positives. The peptide count for remaining peptides was normalized by percentage contribution to the total peptide count in the sample. Enrichment was then calculated as the fold-change in each peptide’s normalized frequency in the selected (gut-bound) sample relative to its frequency in the naïve library so that selected binding peptides could be distinguished from abundant sequences in the unselected library. The remaining peptides were then filtered to exclude target-unrelated binders (using the SAROTUP suite of bioinformatic tools [32–34]), and the predicted stability and hydrophilicity were determined. Stable, hydrophilic gut-binding peptide candidates were retained. This pipeline reduced the 10,546 unique *D. citri* nymph selected sequences with log_2_ fold change ≥ 3 to a final pool of 26 candidate gut-binding peptides. From this pool, we selected 10 enriched peptides based on one of the following criteria: (i) peptides with the highest enrichment during the two rounds of phage selection in *D. citri* nymphs, (ii) peptides with gut binding confirmed, and (iii) peptides with sequence similarity to *C*Las surface proteins.

The selected peptides included eight that bound to the nymph gut (DcNy) and two that bound to adult gut-derived brush border membrane vesicles (BBMV; DcAd), with gut binding confirmed (Table 1; Supplemental Methods, Fig. S1 [35]). Of these peptides, six were hydrophilic, four were hydrophobic, and seven showed strong similarity to exposed *C*Las proteins (Table 1). To confirm peptide binding to the gut for two DcAd and two DcNy peptides, UV crosslinking pull-down assays were used to identify primary binding partners on the *D. citri* gut surface (Table 2; Fig. S2). Aminopeptidase N-like protein was the primary binding partner for DcNy2 and DcAd4, whereas alkaline phosphatase and neprilysin-2-like were the main binding partners for DcNy10 and DcAd3, respectively.

**TABLE 1:** Gut-binding peptides (GBPs) used for this study

Peptide name^*a*^	AA sequence	GRAVY score	Nature	Primary solvent	Selection criteria^*b*^
DcNy1	CTQTNNAYC	−0.771	Hydrophilic	Nuclease-free water	Highly enriched
DcNy2	CIAGKARGC	0.433	Hydrophobic	Dimethyl sulfoxide (DMSO)	SP match; highly enriched
DcNy10	CTFPNTSSC	−0.033	Hydrophilic	Nuclease-free water	SP match
DcNy11	CGMGTAGVC	1.222	Hydrophobic	DMSO	SP match
DcNy12	CSSSLDKAC	0.089	Hydrophobic	DMSO	SP match
DcNy13	CTLKNNAYC	−0.256	Hydrophilic	Nuclease-free water	SP match
DcNy14	CKQSNNAYC	−1.078	Hydrophilic	Nuclease-free water	SP match
DcNy15	CNMSPGRIC	0.067	Hydrophobic	DMSO	SP match
DcAd3	CNNVTHRVC	−0.222	Hydrophilic	Nuclease-free water	Binding confirmed^*c*^
DcAd4	CYPYTNTSC	−0.544	Hydrophilic	Nuclease-free water	Binding confirmed^*c*^
NegP	CAPAPAPAC	0.822	Hydrophobic	DMSO	Not applicable

^*a*^ DcNy peptides isolated from 4th instar *Diaphorina citri *nymphs; DcAd peptides isolated from *D. citri *adults (28).

^*b*^ SP, amino acid sequence match with *C*Las surface protein.

^*c*^ See Fig. S1.

**TABLE 2:** Primary *Diaphorina citri* gut surface-binding partners of selected gut-binding peptides tested for competition with *C*Las^*a*^

GBP	Binding partner	Identifier^*b*^	Mr (kDa)	TSC	Sequest HT score/ −10lgP
DcNy2	Aminopeptidase N-like^*c*^	MCOT21830.0.CT	111	30	131.26
DcNy10	Alkaline phosphatase	MCOT15347.0.CC	62	25	133.05
DcAd3	Neprilysin-2-like	XP_026683286.1	89	6	16.32
DcAd4	Aminopeptidase N-like^*c*^	XP_026679329.1	62	9	20.11

^*a*^ Mr, molecular mass; TSC, total spectrum count.

^*b*^ Binding partners of nymph GBP were identified with reference to the citrusgreening.org database. The amino acid sequences of these two proteins are provided in the supplemental material. The binding partners of adult GBP were identified with reference to a *D. citri*-specific NCBI database.

^*c*^ The two aminopeptidase N-like proteins are the same gut surface proteins: the reference sequence in the Citrus Greening Solutions database is complete.

### GBP treatments reduce *C*Las acquisition in *D*. *citri* nymphs and adults

**Fig 1:**
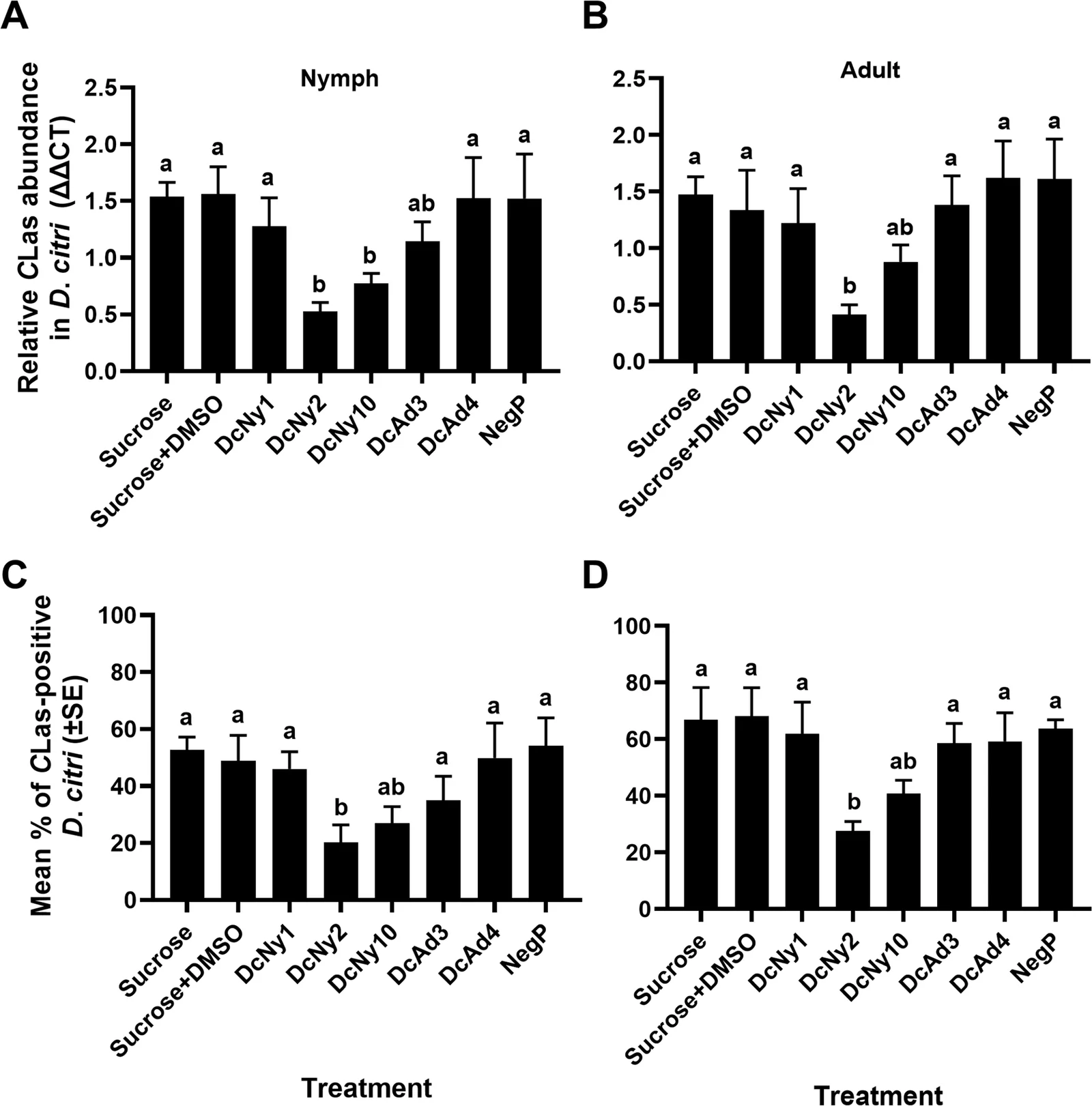
Gut-binding peptides (GBPs) decreased *Candidatus* Liberibacter asiaticus acquisition and infection in *Diaphorina citri*. Panels **A** and **B** show the abundance of *C*Las in nymphs and adults, respectively, following a 48-h acquisition access period on infected *Citrus sinensis*. Panels **C** and **D** depict the overall *C*Las infection rates in nymphs and adults. Data are presented as mean ± SEM of six independent replicates (*n* = 6), with 10 nymphs or adults per replicate. Different letters indicate statistically significant differences (analysis of variance [ANOVA], Tukey’s honestly significant difference [HSD] test, *P* < 0.05).

The 10 selected GBPs were evaluated for their ability to compete with *C*Las for binding to the psyllid gut. To evaluate the impact of GBPs on *C*Las acquisition, *D. citri* nymphs and adults each were fed gut-binding peptide or the negative control peptide (Table 1) and subsequently exposed to flush tissue (i.e., new leaves being produced) from *C*Las-infected citrus trees. NegP was included as a non-gut-binding peptide control to account for the potential effects of peptide feeding and solvent exposure that were unrelated to specific binding of GBPs to the psyllid gut. Significant reductions in *C*Las abundance were observed for specific treatments in both developmental stages (Fig. 1). Among nymphs, treatments with DcNy2 and DcNy10 significantly reduced *C*Las loads relative to controls (*F* = 2.701; *P* = 0.022; Fig. 1A), whereas in adults, only DcNy2 resulted in a significant reduction in *C*Las levels compared with the control group (*F* = 2.369; *P* = 0.041; Fig. 1B).

Infection rates were also significantly reduced following GBP treatment in both nymphs and adults (Fig. 1). The proportion of *C*Las-positive nymphs and adults was significantly reduced by DcNy2 treatment (nymphs: *F* = 1.918; *P* = 0.0474; Fig. 1C; adults: *F* = 2.678; *P* = 0.0226; Fig. 1D). Based on their effects on *C*Las acquisition and infection rates, peptides DcNy2, DcNy10, and DcAd3 were selected for further analysis. Although not significantly different from the controls, DcAd3 was included based on the numerical reduction in *C*Las acquisition by nymphs (Fig. 1A), which is a critical step in the establishment of infection and subsequent transmission. As no significant effects on relative *C*Las abundance or infection incidence were observed for the second set of peptides tested (DcNy11 to DcNy15) (Fig. S3), work on these peptides was discontinued.

### GBP treatments reduce *C*Las abundance in *D*. *citri* adult alimentary canal and salivary glands

**Fig 2:**
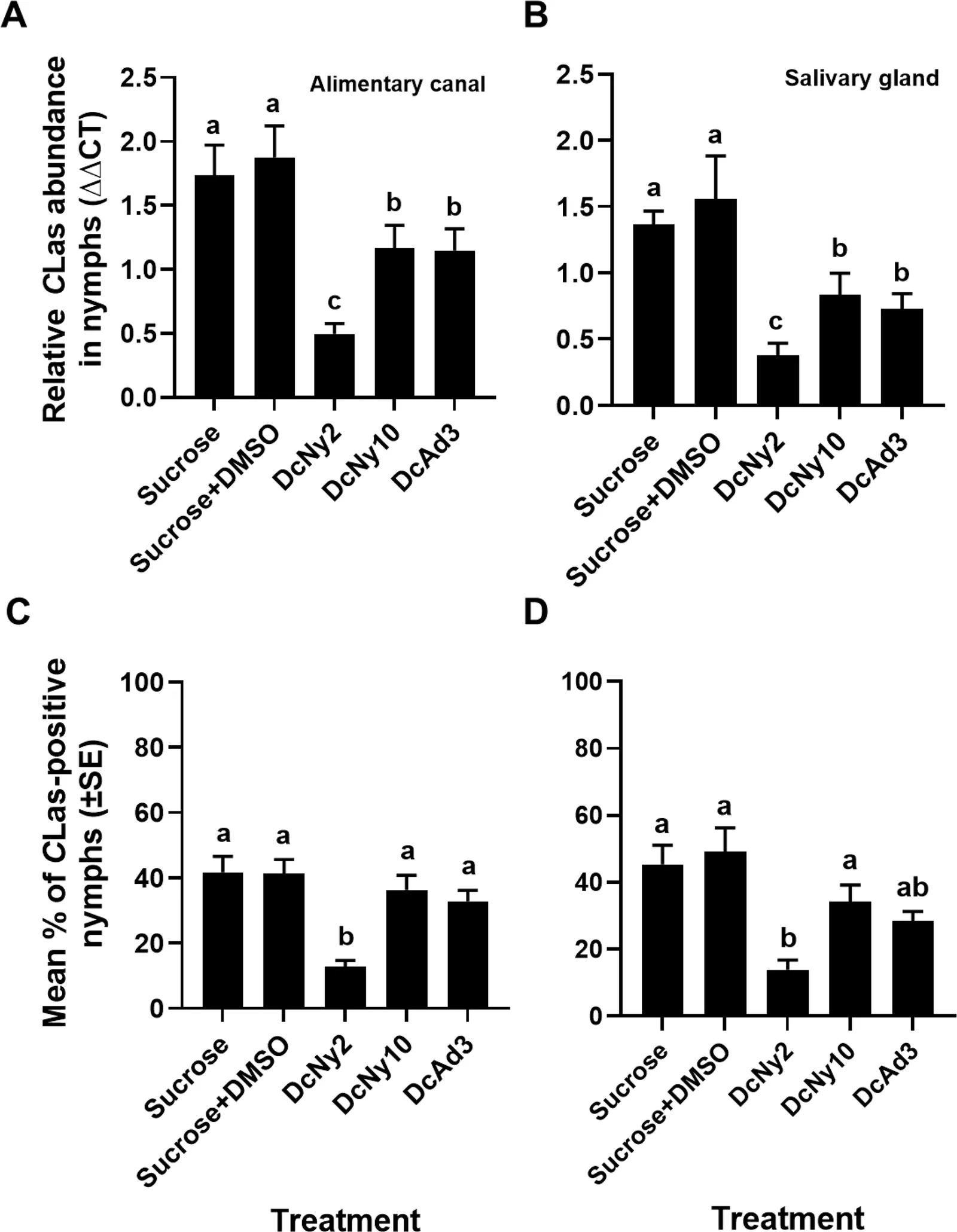
Reduction in *Candidatus* Liberibacter asiaticus abundance and infection incidence in the *Diaphorina citri* alimentary canal and salivary glands. Following feeding on *C*Las-infected plants, nymphs were transferred to *C*Las-free plants for 7 days, by which time they reached adulthood. Panels **A** and **B** show the abundance of *C*Las in the alimentary canal and salivary glands of adults, respectively, following a 48-h acquisition access period on infected *Citrus sinensis*. Panels **C** and **D** depict the overall *C*Las infection rates in the alimentary canal and salivary glands. Data are presented as mean ± SEM of six independent replicates (*n* = 6), with 7–10 adults per replicate. Different letters indicate statistically significant differences (ANOVA, Tukey’s HSD test, *P* < 0.05).

To determine whether the three GBPs of interest block *C*Las *in vivo*, individual adult psyllids from the inoculation assay were dissected and analyzed. GBP treatments significantly reduced *C*Las abundance in both the alimentary canal (*F* = 8.189; *P* = 0.0002; Fig. 2A) and salivary glands (*F* = 7.095; *P* = 0.0006; Fig. 2B). Tukey’s *post hoc* analysis indicated that DcNy2 treatment produced the greatest reduction in *C*Las abundance, while DcNy10 and DcAd3 yielded intermediate decreases, with values falling between those of DcNy2 and the control treatments (Fig. 2A and B). Similarly, infection rates differed significantly among treatments in the alimentary canal (*F* = 10.67; *P* = 0.0001; Fig. 2C) and salivary glands (*F* = 8.497; *P* = 0.0002; Fig. 2D). DcNy2 resulted in the lowest infection rates in both tissues compared with the controls (Fig. 2C and D).

### GBP treatments reduce *C*Las inoculation in *C*. *sinensis* plants

**Fig 3:**
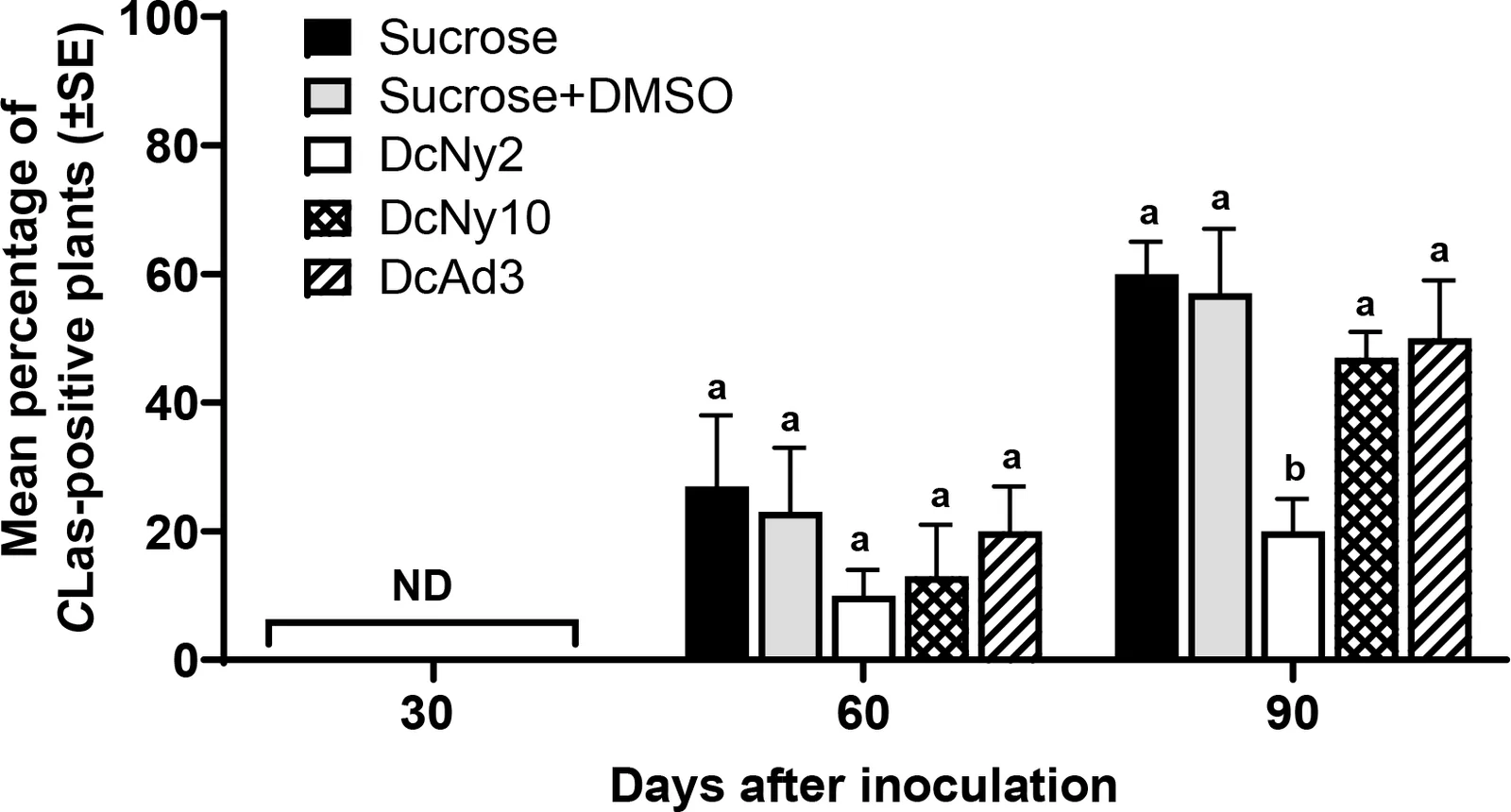
Pretreating *Diaphorina citri* with DcNy2 disrupted subsequent inoculation of *Candidatus* Liberibacter asiaticus (*C*Las). Inoculation was assessed by measuring plant infection at 30, 60, or 90 days following inoculation access periods on initially uninfected *Citrus sinensis*. Data represent the mean ± SEM of *C*Las-positive plants from six replicates, with five plants per replicate. Different letters indicate statistically significant differences (ANOVA, Tukey’s HSD test, *P* < 0.05). ND = not detected.

To assess how acquisition of these peptides affects subsequent transmission of the pathogen, uninfected *C. sinensis* plants were exposed to *C*Las-infected *D. citri* nymphs that had been pretreated with either GBP or a control diet. At 30 days post-inoculation, *C*Las was undetectable in all treatment groups (Fig. 3). By 60 days, plants exposed to DcNy2-treated nymphs exhibited the lowest infection rates, although this difference was not statistically significant (*F* = 0.5747; *P* = 0.6835). However, by 90 days, plants exposed to DcNy2-treated *D. citri* nymphs exhibited significantly lower infection rates compared to other treatments (*F* = 5.422; *P* = 0.0028).

### GBP treatments do not impair *D*. *citri* fecundity and development

**Fig 4:**
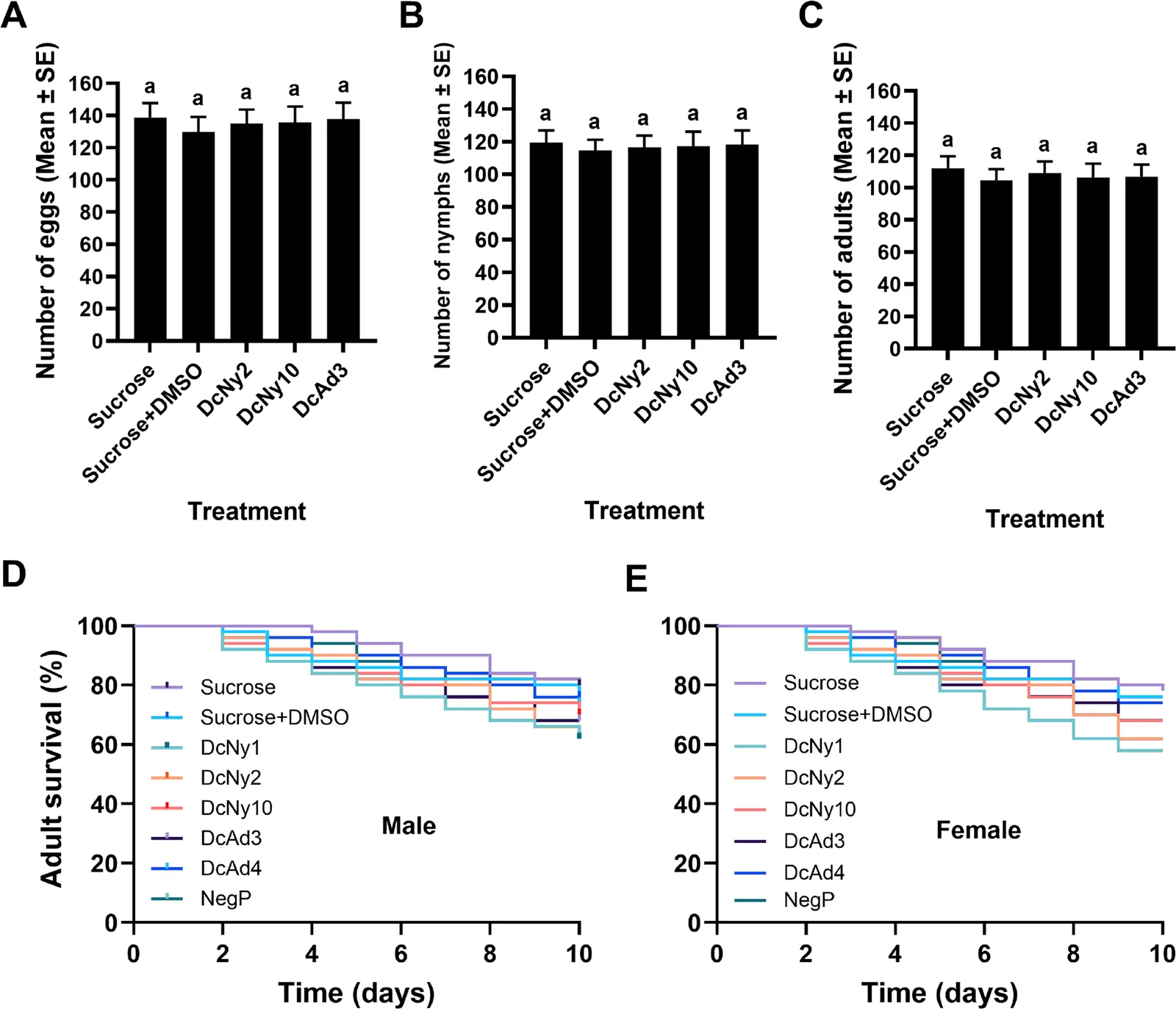
Effects of gut-binding peptides (GBPs) on *Diaphorina citri* fecundity and survival. (**A**) Total egg production, (**B**) total nymph production, and (**C**) percentage of adult emergence. Data are presented as mean ± SEM for 15 females per treatment (*n* = 15). Each female was paired with two males, and the numbers of eggs laid, nymphs produced, and adults emerged were recorded. Treatments sharing the same letter are not significantly different (ANOVA, Tukey’s HSD test, *P* < 0.05). Survival of adult (**D**) male and (**E**) female *D. citri* exposed to GBPs is shown as Kaplan-Meier longevity curves. Each treatment was replicated five times with 10 adults per treatment. Survival curves were compared using the log-rank test (*P* < 0.05).

To evaluate potential fitness costs associated with exposure of psyllids to the three selected peptides, reproductive output and developmental success were monitored across treatment groups. No significant differences were detected in fecundity (*F* = 0.138; *P* = 0.966; Fig. 4A), nymphal production (*F* = 0.057; *P* = 0.994; Fig. 4B), or adult emergence (*F* = 0.147; *P* = 0.964; Fig. 4C) among treatments.

### Impact of GBP treatments on *D*. *citri* adult mortality

No significant differences in adult mortality among treatments were noted on Kaplan-Meier survival analysis. Neither male nor female survival differed across the treatments (males: log-rank test: χ^2^ = 8.422, *df* = 7, *P* = 0.297; Fig. 4D; females: log-rank test: χ^2^ = 9.894, *df* = 7, *P* = 0.195; Fig. 4E).

## DISCUSSION

The motivation for this study was to determine whether *D. citri* gut-binding peptides with or without sequences similar to those of *C*Las surface proteins would compete with *C*Las for binding sites in the psyllid gut. Reduced association of *C*Las with the psyllid gut surface is expected to reduce psyllid infection and subsequently limit transmission of the pathogen to citrus plants. Based on data presented herein, we conclude that peptide DcNy2 effectively reduced *C*Las association with the gut, leading to significant reductions in both pathogen abundance and infection incidence in adult and nymphal *D. citri* and ultimately decreasing inoculation of *C*Las into healthy citrus hosts. Because nymphs are more efficient at acquiring *C*Las due to prolonged feeding and immature immune responses (8), the inhibition observed in this life stage is particularly noteworthy. Tissue-specific pathogen quantification further indicates that DcNy2 disrupted bacterial passage into the hemolymph and salivary glands, which are key barriers in the circulative-propagative transmission pathway (24). Importantly, GBP treatments selectively target *D. citri* gut surface proteins without compromising key fitness traits, supporting a specific, pathogen-interference mode of action.

### Blocking pathogen infection of vector species

Reagents that bind to the psyllid gut and interfere with *C*Las-gut association are of particular interest because they target the earliest stage of vector infection. DcNy2 likely interferes with bacterial adhesion or entry at the gut epithelium, a strategy that is consistent with previous work showing that peptide-based approaches can disrupt *C*Las acquisition by *D. citri*. For example, Merfa et al. (23) demonstrated that peptides derived from the *C*Las outer membrane protein OmpA reduced *C*Las acquisition by adult *D. citri* when delivered through artificial diet or *in planta*. Whereas that approach targeted a candidate *C*Las surface protein predicted to participate in host recognition or adhesion, the present study identified peptides that bind directly to *D. citri* gut surface proteins and interfere with *C*Las acquisition, tissue infection, and subsequent inoculation. Similar interference strategies have been successfully applied in other vector-pathogen systems. For example, *Xylella fastidiosa* Wells uses afimbrial adhesins to bind to carbohydrates in the foregut of the leafhopper vector, *Graphocephala atropunctata* (Signoret) (36), and disrupting this adhesion with competitive molecules significantly reduces bacterial acquisition and transmission (26, 37). Similarly, circulative plant virus-vector interactions have been successfully disrupted using recombinant viral proteins or peptides (22, 38). In the case of tomato spotted wilt virus, a soluble form of its envelope glycoprotein binds to the midgut of the thrips vector, *Frankliniella occidentalis* Pergande, reducing both viral loads and transmission to healthy plants (25, 39). Likewise, a 12-amino acid peptide GBP3.1 competes with pea enation mosaic virus for receptor binding (40), thereby impeding viral entry into the aphid vector (27). Together, these examples illustrate the broader feasibility of peptide-based strategies for disrupting pathogen acquisition and reducing vector competence across diverse vector-pathogen systems.

### Gut protein binding by *C*Las

The identification of binding partners for four of the peptides confirmed their capacity to bind psyllid gut surface proteins. Notably, the aminopeptidase N and alkaline phosphatase proteins bound by DcNy2 and DcNy10, respectively, are the two most abundant proteins on the gut surface of *D. citri* nymphs (41). Pathogens that exploit highly abundant proteins on the gut surface of their respective hosts increase the likelihood of infection (22). The use of multiple receptors rather than a single gut surface protein that could be rapidly modified or downregulated would also favor successful infection. Indeed, redundancy among gut surface proteins that mediate the toxic action of bacterial pesticidal proteins is well documented (42, 43).

The potential involvement of nine specific gut surface proteins in mediating *C*Las entry into the gut of *D. citri* was recently investigated by use of gene silencing (44). Among the proteins evaluated were aminopeptidase N-like (APN; MCOT21830.0.CT) and alkaline phosphatase (ALP), which were the same proteins identified in the current study as binding partners for DcNy2 and DcNy10, respectively (Table 1). Both ALP and APN emerged as promising RNAi targets for disrupting vector competence, with the authors concluding that ALP is “likely involved” and APN “potentially involved” in *C*Las binding to the *D. citri* gut. Together, these gene silencing results along with the peptide-mediated interference of *C*Las association with these proteins reported here strongly support ALP and APN as leading candidates for *C*Las receptor proteins in the psyllid gut.

### Delivery systems for DcNy2 to reduce *C*Las transmission in the field

The practical application of DcNy2 to reduce *C*Las transmission in the field will require an effective delivery system capable of ensuring sufficient peptide accumulation in the psyllid gut to interfere with *C*Las binding. Both transgenic and paratransgenic approaches offer potential solutions. Transgenic citrus plants have already been engineered to deliver bacterial pesticidal proteins targeting *D. citri* (45) and antimicrobial peptides targeting *C*Las (46). However, whether such systems can express DcNy2 at levels sufficient to reach the gut and block the rapid and efficient *C*Las infection process remains to be empirically tested. The use of a phloem-mobile peptide carrier to facilitate vascular transport and ingestion by psyllids has been demonstrated (47) and may further enhance appropriate delivery.

Paratransgenesis provides an alternative strategy through the engineering of insect symbionts to deliver antimicrobial or gene-silencing molecules directly within the vector. Proof-of-concept for this strategy was first demonstrated in *Rhodnius prolixus* Stål, where engineered *Rhodococcus rhodnii* expressing cecropin-A suppressed *Trypanosoma cruzi* transmission (48). Similar successes have been achieved in mosquitoes and tsetse flies using *Wolbachia* and *Sodalis* symbionts, respectively (49, 50), and in honeybees, where *Snodgrassella alvi* was engineered to deliver viral gene-silencing RNAs directly into the gut (51). These studies demonstrate that symbiont-mediated delivery of antimicrobial peptides, antibodies, or RNAi constructs can substantially reduce pathogen transmission. In *D. citri*, paratransgenic approaches are in development, with resident symbionts such as *Wolbachia*, *Profftella*, and *Carsonella* as potential candidates for effector expression. Importantly, the stable maternal transmission of these symbionts suggests that engineered strains could persist across psyllid generations (52–54).

Neither transgenic nor paratransgenic DcNy2 delivery systems will provide a stand-alone solution to citrus greening, but both represent powerful tools within a multipronged, integrated disease management framework. Transgenic citrus-expressing GBPs could limit *C*Las acquisition at the plant interface, while engineered symbionts releasing GBPs within the *D. citri* gut and propagated vertically through populations could provide sustained interference with transmission. When integrated with established practices such as removal of infected trees, biological control of *D. citri*, and protection of clean nursery stock, these strategies have the potential to help restore citrus production in severely impacted regions such as Florida and enhance quarantine protection in areas where citrus greening remains limited.

## MATERIALS AND METHODS

### Insect culture and plant source

*D. citri* nymphs and adults were sourced from *C*Las-free insect colonies originally established on *Citrus sinensis*, *Murraya paniculata*, and *Bergera koenigii* at the Citrus Research and Education Center (CREC) in Lake Alfred, FL (55). Insects were reared on plants at 25 ± 2°C, 60 ± 5% relative humidity, with a 14-h:10-h (light-dark) photoperiod. To ensure the colonies remained *C*Las-free, both the psyllids and nursery-sourced *C. sinensis* plants were tested every 2–3 months using quantitative polymerase chain reaction (qPCR) (56). *C*Las-positive plants, confirmed by qPCR, were maintained separately after graft inoculation with infected budwood. All insect and plant cultures were maintained without insecticide exposure.

### Selection and testing of gut-binding peptides

Gut-binding peptides (GBP) were isolated by screening the Ph.D.-C7C library (New England BioLabs, Ipswich, MA, USA) against either *D. citri* adults as described previously (28) or against 4th-instar nymphs. To select phage displaying peptides that bind to the gut of *D. citri* nymphs, 100 nymphs were fed on a 30% sucrose solution containing 10^11^ pfu/mL of phage by using the wipe-based feeding method (57). Nymphs were fed on phage solution for 48 h with fresh phage solution provided after 24 h to ensure phage viability. Guts were then dissected under a microscope in TBS (pH 7.5) containing 1% BSA, cut into small pieces (Fig. S4), transferred to a 1.5-mL microcentrifuge tube, and washed three times in TBS with 0.1% Tween-20 (TBST) for 5 min at 4°C under gentle rotation. The tissues were then centrifuged at 1,000 × *g* for 2 min for removal of unbound phages. Elution of bound phage, titering, phage amplification, and phage DNA extraction along with bioinformatics analyses were performed as previously described (28). Two rounds of phage enrichment were conducted for *D. citri* nymphs.

To confirm gut binding of selected peptides from the phage library screen in adult *D. citri*, peptide-linker-mCherry fusion proteins containing an (AP)5 linker were generated, expressed, and purified as described previously (31). The purified peptide-mCherry fusion proteins were fed to *D. citri* adults that had been starved overnight, and dissected guts were examined with a fluorescence microscope (see Supplemental Methods).

Peptides for further study were selected based on one of three criteria. First, peptides with the highest enrichment during the two rounds of phage selection in *D. citri* nymphs; second, peptides with confirmed gut binding; third, peptides with sequence similarity to *C*Las surface proteins. For sequence comparisons of adult (DcAd) and nymph (DcNy) gut-binding peptides, only the 7-residue insert (excluding the flanking cysteines) was considered. To identify peptides with similarity to *C*Las surface proteins, peptides were locally aligned to the extracellular segments of a curated set of *Candidatus* Liberibacter asiaticus (*C*Las) surface proteins (58), whose extracellular domains were defined from membrane-topology annotations and whose residue-level surface exposure was assessed by sequence-based solvent-accessibility predictions. Percent identity was computed as (number of identical positions ÷ 7) ×100. GBPs with similarity to *C*Las surface proteins selected for testing for competition with *C*Las in the psyllid gut satisfied all of the following criteria: (i) enrichment with log2 fold-change ≥3 in at least one biopanning round compared to the naïve reference library; (ii) ≥70% identity to a *C*Las extracellular segment (i.e., ≥5/7 identical residues); (iii) presence of at least one uninterrupted 5-residue stretch identical to the matching *C*Las region; and (iv) all matched residues mapped to the exposed surface of the extracellular domain.

The selected GBPs along with a non-gut-binding peptide (NegP) used as a negative control were synthesized by Genemed Synthesis Inc. (San Antonio, TX, USA). Peptide hydrophobicity and hydrophilicity were evaluated using the grand average of hydropathy (GRAVY) score (http://www.gravy-calculator.de/). Hydrophilic peptides were dissolved in nuclease-free water (Life Technologies, TX, USA), while hydrophobic peptides were dissolved in dimethyl sulfoxide (DMSO; Sigma Aldrich, MO, USA). An artificial diet was prepared for delivering treatments to psyllids, consisting of 17% (wt:vol) sucrose and sucrose +DMSO solution in nuclease-free water, supplemented with 0.1% green food dye and 0.4% yellow food dye (McCormick & Co., Inc., Hunt Valley, MD, USA). The final DMSO concentration in the artificial diet was 0.625%.

### Identification of peptide-binding partners

To confirm binding to gut surface proteins, UV crosslinking pull-down assays were conducted with selected peptides as described previously (59). For the DcAd peptides (selected from *D. citri* adults), the UV crosslinking pull-down assay was performed using BBMV prepared from whole psyllids. The binding partners of DcNy peptides (selected from *D. citri* nymphs) were determined by using a modified *in vivo* UV crosslinking pull-down assay in which peptides were fed to *D. citri* nymphs, and the dissected guts were UV-irradiated rather than using BBMV. Briefly, *D. citri* 4th-instar nymphs (~50 nymphs per peptide) were fed on 25% sucrose solution containing 300 µg/mL of Bpa- and biotin-labeled peptide for approximately 36 h using the sandwich feeding assay (60). Negative controls consisted of nymphs fed on the peptide without UV treatment or nymphs fed on sucrose solution only. The guts were dissected under a microscope, transferred to a cold 16-well plate blocked with 0.5% BSA in 1× PBS (pH 7.6), and exposed to UV 365 nm on ice for 9 min or 2 J of energy. The guts were then homogenized in 1× TBS pH 7.6, 1% SDS, 1× Protease Inhibitor (Thermo Scientific, Rockford, IL, USA), and 1 mM PMSF, and the homogenates were incubated at 70℃ for 30 min followed by centrifugation at 16,000 × *g* for 15 min. The following procedures were performed as described previously for the UV crosslinking pull-down assay using BBMV (59). The UV crosslinked products were resolved in 4%–12% SDS-PAGE gels, and the gel lanes containing the peptide-binding partners or controls were excised in two equal segments and sent for liquid chromatography-tandem mass spectrometry (LC-MS/MS) (59). Sample preparation and LC-MS/MS were performed as previously described (41). To confirm successful crosslinking between labeled peptides and *D. citri* gut surface proteins, we performed western blots with UV-irradiated products. Proteins were detected using streptavidin HRP (1:5,000, Thermo Scientific), followed by a chemiluminescent substrate.

For identification of proteins bound by GBP, Scaffold (v4.11.1; Proteome Software Inc.) or PEAKS X (Bioinformatics Solutions Inc.) was used to validate MS/MS-based peptide and protein identification. A *D. citri*-specific NCBI database was used as a reference for identification of adult GBP-binding partners, while a protein database from the Citrus Greening Solutions website (citrusgreening.org) was used for identification of nymph GBP-binding partners. The acceptance threshold for peptide identification was a >95% probability by the Scaffold Local FDR algorithm. For protein identification, the threshold was >99.9% probability with at least one unique peptide. Putative peptide-binding partners were defined as follows: (i) proteins with at least one unique peptide and ≥2 total spectral matches (TSC); (ii) proteins unique to the UV-crosslinked lane or exhibiting at least 3-fold higher TSC in the UV-crosslinked lane compared to the non-UV lane; and (iii) proteins predicted to localize to the plasma membrane or to possess a GPI anchor. Candidate peptide-binding partners were assessed for subcellular localization (BUSCA, DeepLoc), presence of a GPI anchor (PredGPI), transmembrane domains (TMHMM), and signal peptides (SignalP 4.1). Each predicted surface protein was then manually verified in the UniProt database to confirm subcellular localization and cross-checked against the gut surface proteome of *D. citri* (41).

### *C*Las acquisition assay

To evaluate the ability of GBPs to inhibit *C*Las acquisition, 1- to 2-day-old adult *D. citri* were exposed to GBP treatments using a membrane-feeding bioassay (57) for 24 h. Control groups were fed a sucrose + DMSO solution containing a non-gut-binding peptide (NegP), which served as a negative control. A GBP concentration of 100 µg/mL was used to assess potential effects on *C*Las acquisition. For *D. citri* nymphs, peptides were delivered using a novel method referred to as the immobilized droplet feeding assay (IDFA; Fig. S5). Briefly, *C*Las-free 3rd- to 4th-instar nymphs were immobilized on 1 × 1 cm sticky paper squares (Post-it, 3M, Saint Paul, MN) affixed to plastic lids. A 0.14–0.2-µL droplet of peptide solution (100 µg/mL) was applied to the ventral thorax using a borosilicate glass capillary (WPI, Sarasota, FL), and nymphs were allowed to feed for 5 min. Following feeding, nymphs were transferred to *C*Las-infected *C. sinensis* plants with young flushing leaf tissue. Flushes were randomly assigned among treatments within each experiment such that any variation in *C*Las abundance among flushes would be distributed across treatments. To limit movement, the flush was enclosed using a filter paper cone (P5, Fisher Scientific, Pittsburgh, PA, USA). For the acquisition assays, groups of 10 adults or nymphs were fed GBP treatments and then caged on *C*Las-positive *C. sinensis* sweet orange plants inside mesh enclosures for a 48-h acquisition access period (AAP). After the AAP, insects were collected and preserved in 80% ethanol at −20°C for subsequent *C*Las detection via qPCR, following the protocol as previously described (7). Each treatment was replicated six times for each developmental stage.

### *C*Las inoculation assay

To quantify the efficacy of GBPs in reducing *C*Las transmission to susceptible plants, *C*Las-free 3rd- to 4th-instar nymphs were fed GBP solutions (100 µg/mL) or control treatments using the IDFA method described above. Following treatment, nymphs were transferred to randomly assigned *C*Las-positive flushing leaves for a 48-h AAP. After the AAP, groups of ten nymphs per treatment were transferred to *C*Las-free *C. sinensis* plants enclosed in mesh cages. After a 7-day inoculation access period (IAP), the resulting adults were collected and transferred to the laboratory for *C*Las detection. The effect of GBP treatment on *C*Las transmission was assessed by quantifying bacterial loads in the insect alimentary canal, salivary glands, and in leaf tissue from inoculated plants. Dissection of adult psyllids followed the procedure described in reference 61. Each adult psyllid was dissected into two parts: (i) the salivary glands, which remained attached to the head for easier handling with the stylet carefully removed (Fig. S6A); and (ii) the alimentary canal, which included the filter chamber, midgut, and Malpighian tubules, separated from the rest of the body (Fig. S6B). Leaf samples were collected from inoculated plants at 30, 60, and 90 days post-feeding to monitor *C*Las infection. Each treatment was replicated five times, and the entire experiment was repeated on six separate occasions.

### Effect of GBPs on adult *D. citri* fecundity and development

To assess the fecundity of GBP-treated *D. citri*, 2- to 3-day-old adult psyllids were exposed to GBPs using the membrane feeding assay described previously. Briefly, groups of 10 males or 10 females were placed individually in vials and fed for 24 h on membrane sachets containing either GBP (100 μg/mL) or control treatments. After feeding, a single female was paired with two males and transferred to a *C*Las-free *C. sinensis* plant with young flushing tissue to allow mating and oviposition. Eggs laid per female were counted every 2 days for up to day 6 and additionally on day 7 under a stereomicroscope (SZX2-ILLK, Olympus Corporation, Japan). To facilitate continuous oviposition, adults were transferred to fresh flush tissue throughout the observation period. Nymph hatch rates were recorded daily for 7–14 days, and adult emergence from nymphs was recorded daily at 14–20 days after the start of the experiment. Each treatment was replicated 15 times, with one female psyllid per replicate.

### Effect of GBPs on adult psyllid survival

To evaluate the effect of GBPs on adult *D. citri* survival, 1- to 2-day-old *D. citri* adults were separated by sex under a stereomicroscope and fed on either GBP (100 μg/mL) or control treatments by membrane feeding bioassay. After 24 h, the treated adults were transferred onto *C*Las-free *C. sinensis* plants placed in mesh cages under controlled greenhouse conditions as described above. Survival of GBP-treated and untreated *D. citri* adults was then monitored over 10 days by observing 10 adults of each sex per treatment. Each treatment was replicated five times.

### *C*Las isolation and determination of relative abundance

To quantify the relative abundance of *C*Las, DNA from insect and plant samples was isolated using the DNeasy Blood and Tissue or DNeasy plant kits (Qiagen, Valencia, CA, USA), according to the manufacturer’s instructions. The isolation of DNA from the *D. citri* alimentary canal and salivary glands was performed by the cetyltrimethylammonium bromide (CTAB) method (62). The concentration of the extracted DNA was measured using a NanoDrop 2000 spectrophotometer (Thermo Fisher Scientific Inc., Lafayette, CO, USA). The DNA was then adjusted to specific concentrations: 10 ng/μL for plant DNA and 15 ng/μL for *D. citri* DNA, to facilitate quantitative real-time PCR analysis targeting the *C*Las 16S rDNA.

Plant and psyllid extracts were amplified using probe-primer sets targeting internal control sequences specific to plant (cytochrome oxidase) or *D. citri* (insect wingless) gene regions (Table S1) (56, 63). The qPCR assays were conducted in duplicate with a QuantStudio 6 Flex Real-Time PCR System (Applied Biosystems, Waltham, MA, USA). For plants, each reaction mixture was composed of 12.5 μL of PerfeCTaq PCR ToughMix (Quanta BioSciences Inc., MA, USA), Low ROX (Quanta BioSciences Inc., MA, USA), 3 and 3.75 μL of *C*Las and COX primers, respectively, 3.75 μL of each probe, and 1 μL of the DNA template (10 ng) in a final volume of 27.75 μL. For *D. citri*, each reaction mixture was composed of 12.5 μL of PerfeCTaq PCR ToughMix, 3 μL of each primer, 3 μL of each probe, and 1 μL of the DNA template (15 ng) in a final volume of 25.5 μL. Final primer concentrations were 0.2 μM for *C*Las, wingless, and COX. Final probe concentrations were 0.1 μM for *C*Las, wingless, and COX. Real-time PCRs consisted of an initial denaturation step of 95°C for 10 min followed by 40 cycles of 95°C for 15 s for cytochrome oxidase and 60°C for 60 s for wingless. Plates containing plant or insect samples included a no template control (NTC), a positive control (*C*Las DNA in DNA extractions), and a negative control (no *C*Las DNA in DNA extractions). Reactions were considered positive for either target sequence if the cycle quantification (CT) value was ≤36 (7).

### Statistical analysis

The effects of GBP treatments on *C*Las acquisition and infection rates in adult and nymphal *D. citri*, as well as on *C*Las inoculation, fecundity, nymphal count, and adult emergence, were evaluated using analysis of variance (ANOVA) followed by Tukey’s Honestly Significant Difference (HSD) test for multiple comparisons. Percentage data were arcsine square-root-transformed prior to testing for normality and homogeneity of variance using Shapiro-Wilk and Brown-Forsythe tests, respectively. Statistical analyses were conducted using transformed data, whereas untransformed percentages are presented in figures for clarity. Survival rates were estimated using the Kaplan-Meier method (64) and statistically compared using the log-rank test (Mantel-Cox). Statistical significance was defined as *P* < 0.05. All statistical analyses and figure generation were performed using GraphPad Prism 6 (GraphPad Software, San Diego, CA, USA) (65).

## Data Availability

All data are provided within the article and its supplemental material.

## SUPPLEMENTAL MATERIAL

Supplemental material aem.00577-26-s0001.pdf Supplemental methods, Fig. S1 to S6, supplemental sequences, and Table S1.

Dataset S1 aem.00577-26-s0002.xlsx Raw data and statistical analyses.

ASM does not own the copyrights to Supplemental Material that may be linked to, or accessed through, an article. The authors have granted ASM a non-exclusive, world-wide license to publish the Supplemental Material files. Please contact the corresponding author directly for reuse.
