# Supplementary material for: A peptide that binds the Asian citrus psyllid gut reduces transmission of the citrus greening pathogen Candidatus Liberibacter asiaticus
Source: Appl Environ Microbiol. 2026 Sep 4;92(10):e00577-26. doi: 10.1128/aem.00577-26 (PMC13688861; doi:10.1128/aem.00577-26)
Supplement: Supplementary file 1 [file aem.00577-26-s0001.pdf]

Supplementary Information

Supplementary Methods

Confirmation of gut binding of DcAd peptides. To confirm whether selected peptides bind to the *D. citri* gut, purified peptide-mCherry fusion proteins were fed to adult *D. citri* that had been starved overnight and dissected guts were then examined by fluorescence microscopy. Approximately 15 four-day old psyllids per replicate were fed overnight on a 30% sucrose solution containing 0.1% yellow and 0.4% green food dye, supplemented with 150 µg/ml peptide-linker-mCherry. Control insects were fed sucrose solutions containing either linker-mCherry (lmCh) or mCherry alone (mCh). After overnight feeding, guts were dissected in 1x Tris buffered saline - TBS pH 7.4 containing 0.1% Tween (TBST), incubated in True Black blue quencher to reduce autofluorescence, and washed three times with distilled water. Guts were mounted on slides, and mCherry fluorescence was observed under a microscope (Zeiss Axio Imager A2, Zeiss, Germany) with an exposure time of 1500 ms at 17.6% intensity. The same microscope settings were used for all guts and treatments.

To quantify fluorescence intensity across treatments and controls, corrected total fluorescence (CTF) was calculated using ImageJ [1] as follows: CTF = integrated density – (fluorescence of selected gut x mean background fluorescence). Statistical differences in CTF values between the lmCh control and peptide fusions were evaluated by Student`s *t*-test ($P < 0.05$).

Reference cited:

1. Abràmoff MD, Magalhães PJ, Ram SJ, Image processing with ImageJ. *Biophotonics international* **11**(7):36-42 (2004).

Supplementary Information

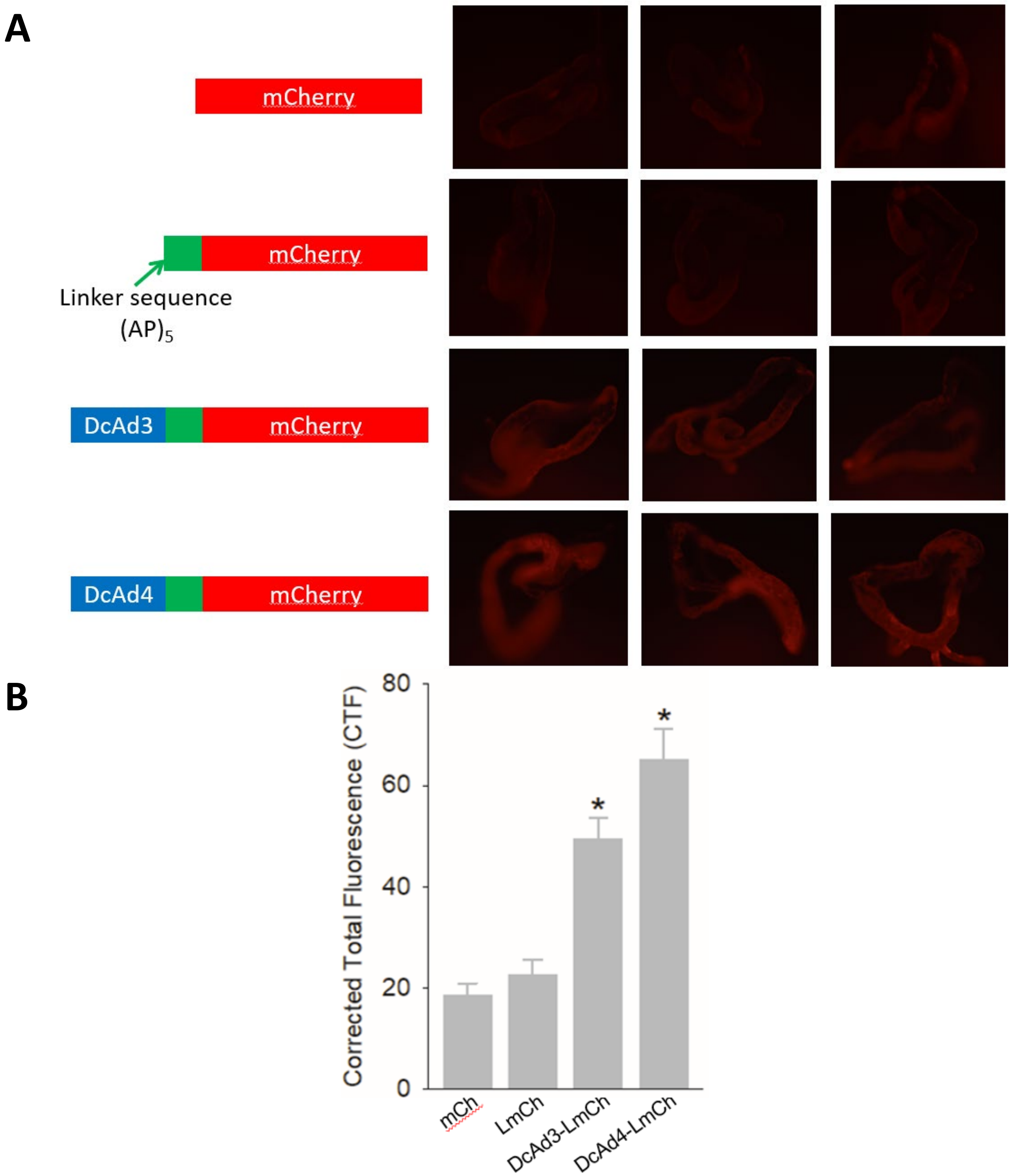

Supplementary Figure S1: Confirmation of peptide binding to the gut of adult *D. citri*. A) Schematic diagram of peptide-linker-mCherry fusions with images showing fluorescence in the gut of adult *D. citri* for the two test peptides fusions, but not the control treatments. B) Corrected total fluorescence (CTF) of guts from *D. citri* adults fed on mCherry (mCh), Linker-mcherry (LmCh) and peptide-Linker-mCherry proteins (DcAd3-LmCh, DcAd4-LmCh). Average and standard error are shown. Asterisks above error bars indicate significant difference relative to the linker-mCherry control according to Student`s *t*-test ($P < 0.05$).

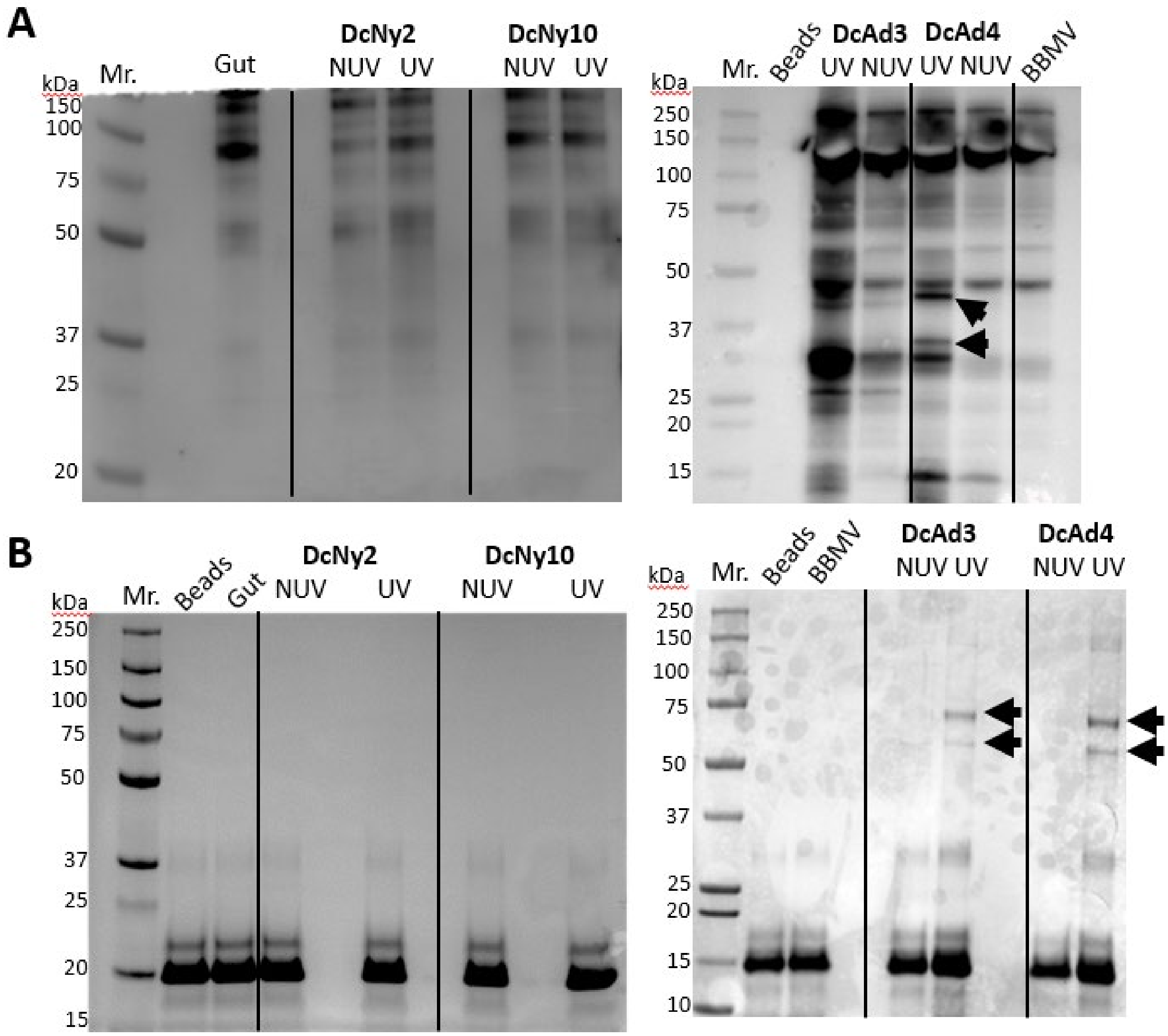

Supplementary Figure S2: UV-crosslinking pulldown assays with DcNy2, DcNy10, DcAd3 and DcAd4. A) Western blot with peptide detection using Streptavidin HRP. Comparison of UV and NUV lanes for each peptide revealed unique bands in some cases (indicated by arrows) that were also absent from the gut or BBMV reference lanes. B) Coomassie stained SDS PAGE gels showing enrichment of proteins (arrows) in some UV irradiated lanes relative to the no-UV treatment and the gut or BBMV reference samples. While western blots and Coomassie stained gels are limited in their sensitivity, peptide binding and subsequent UV-crosslinking to specific gut surface proteins is revealed by mass spectrometry. Mr, Molecular mass marker; NUV, no-UV treatment; BBMV, brush border membrane vesicles.

Supplementary Information

Supplementary Sequences: Amino acid sequences of *Diaphorina citri* aminopeptidase N-like and alkaline phosphatase identified as peptide binding partners from the citrus greening database https://citrusgreening.org/ftp/annotation/MCOT/ as reported in Table 2 of the manuscript.

>**MCOT21830.0.CT | Aminopeptidase N** | Similar to E2BB79 | *** | PANTHER PTHR11533 | PANTHER PTHR11533:SF193 | Pfam PF01433 | Pfam PF11838

MKPVFGCLLLAVVIGHISAIPFDDRLFGAQLKDGETGAVPVMTKFRLPTDLKPTGYEVHV
DPDLAKAEFAGTVKITLTASHSTNFITLNAKYIKLPSAVPVAVTRINTNRPTSTIPVKSSVLD
EANEQLIIELDRALSPKSTYVVQIAYTGRLGSDMSGFYLSRYTNSSGAEKLLASTQFEPTG
ARRAFPCFDEPNYKTPFVIKINRHRHQKAISNMPVSTSVEIANSDKVTDTFQQSPPMPTY
LVGFAVFEFNSLSDKDGKFRVWGREDVVKTQGQYIFDKGPKILAALSDYMGFDYYKML
PKMDLVAVPDFDAGAMENWGMNTYREVYLFLDPSRTKKTRIDDVVSTVTHEFSHQWFG
DLVTPATWNSAWLNEAFATFFEYAGTHWVEPTWRMEDLFVVEQVQVAFNSDLKGSHA
MTSETTTPDSISETFDHIIYNKGGSVLRMIENVLTPTVFQNALQDYLRNGADLGGTVEPA
NLWDAFDEVLTEIGGAQYLPPNSTSITGFVDQWVNKPGFPLVTFSRNYSSKVVTVTQQRF
LVEKADRDADKTNWTIPLTYTTKTNANFSSRANLFWFDRFKPSVQFSIPENNDWLIANL
KEVGFYRVNYDAQNWALIAAQLSSNPAQIHLLNRAQILDDAFTLARADLVPYEAALELT
RYLKTEKDFIPWYAVWSHIRDLLHTYAQTEVANQMKTYFIDRLEGIFNELGLEDKSTEEN
MSKLGRSSISLWICNLGHSGCYNQAVAEFRTWIRAPEKSNIPPNIKDAVYCMGIKYGSGEE
WTRLWQYYLTTDLASEKIAVMYALGCSQNETVLYDYLSKITDPKSGIRSQDHYIVIRGVL
GERPGLRAGLKFLKDKTHSLLEEVKKDELSNAGENLKSIISTVGAGISSKDELNQFTSILT
GYQSNPTIGASINKTLTHIQERFTRSANHQKALEKWFGANTPSTTPSPTTPSQSPLPTHPTS
HPTHATTQKPSGATGLRQFAPSVMVATALVVLVRFGQS

>**MCOT15347.0.CC | Alkaline phosphatase** | Similar to A0A067QT98 | *** | PANTHER PTHR11596 | Pfam PF00245

MVTMWRGCILFFCVLVITVHQGLSRNADDDDDTGMHDFSQFRDYRVGADGGGGGNGG
ASNINEQEKQNKFWIDSAINTLISRTHLQPNTKKAKNIILFLGDGMGVSTHTAARIYQGQL
NGQNGEENSLNFEKFPYTGLSKTYCVDSQVADSACTSTAYTNGVKGSIGTLGLNGRVLY
RDCKGSKDNTTHTESIIHWAIKAGKSTGVVTTTRITHASPAGTYSHSANRDWEADTDRE
ASVDCQDIASQLIKNHPGHMINVLLGGGLKKFTLKKDGGQRLDEDLLLAYERMRQHHN
QTYKIVKTKQELAAVNASTKFLLGLFADSHLEYEVDKQVDKNTKVKDQPSLTEMTQKAI
EVLSKNQNGFYLFVEGGKIDIGHHGNKAKKALTETIEFDNAIKKALQITNPDETLIVVTA
DHSHTFTIAGYPDRGNKILERVRYDGHDLLGLDKLPYLTLGYNNGPGFRPAERNGYRHD
INLDKTDDKDYRIPSGIPLEYETHGGEDVAVFSNGPWAHLLVGNFEQNYIPIVMAYAARIG
PAKDVPLVSAAVSAGPTETLLIAVVSLVVCKFLAKYSK

Supplementary Information

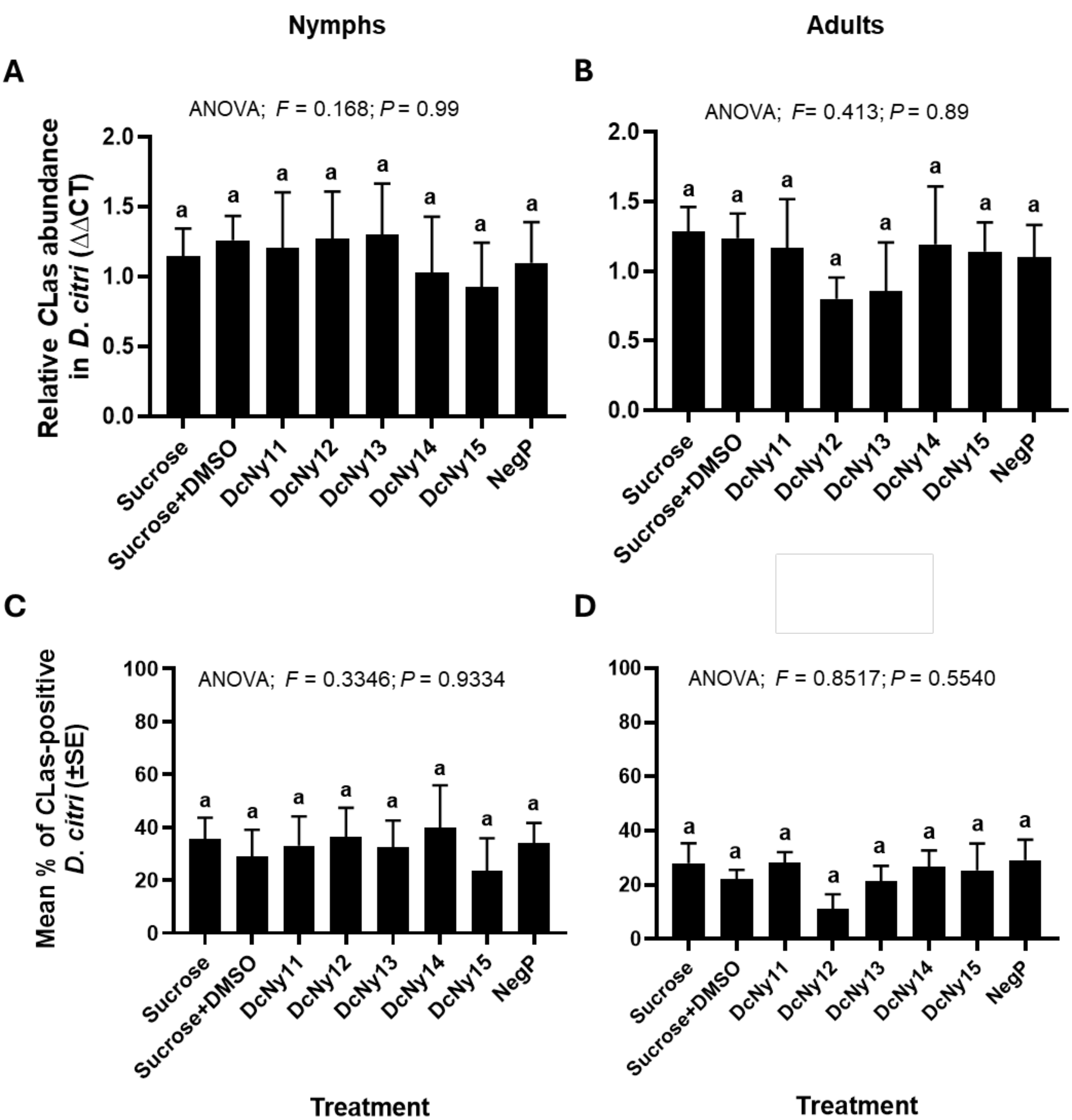

Supplementary Figure S3: Effects of gut binding peptides (GBPs) on *Candidatus* Liberibacter asiaticus acquisition and infection in *Diaphorina citri.* Panels A) and B) show the abundance of *C*Las in adults and nymphs, respectively, following a 48-hour acquisition access period on infected *Citrus sinensis*. Panels C) and D) depict the overall *C*Las infection rates in adults and nymphs. Data are presented as mean ± SEM of six independent replicates (n = 6), with 10 nymphs or adults per replicate. Different letters indicate statistically significant differences (ANOVA, Tukey's HSD test, $P < 0.05$).

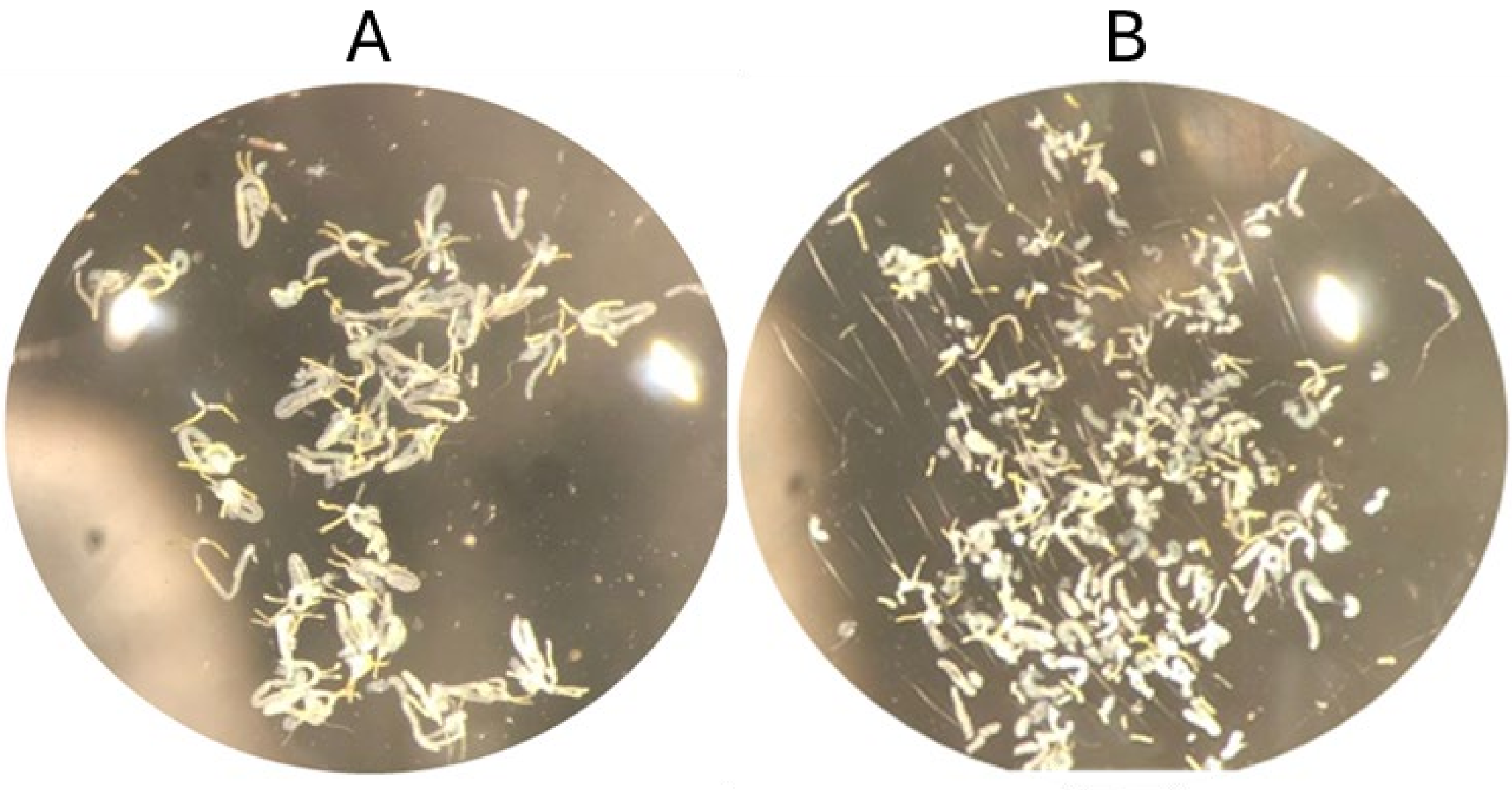

Figure S4. Phage display screening against *Diaphorina citri* nymphs. A) Intact nymph guts containing bound and unbound phages. B) Guts cut into small pieces before washes for removal of unbound phages.

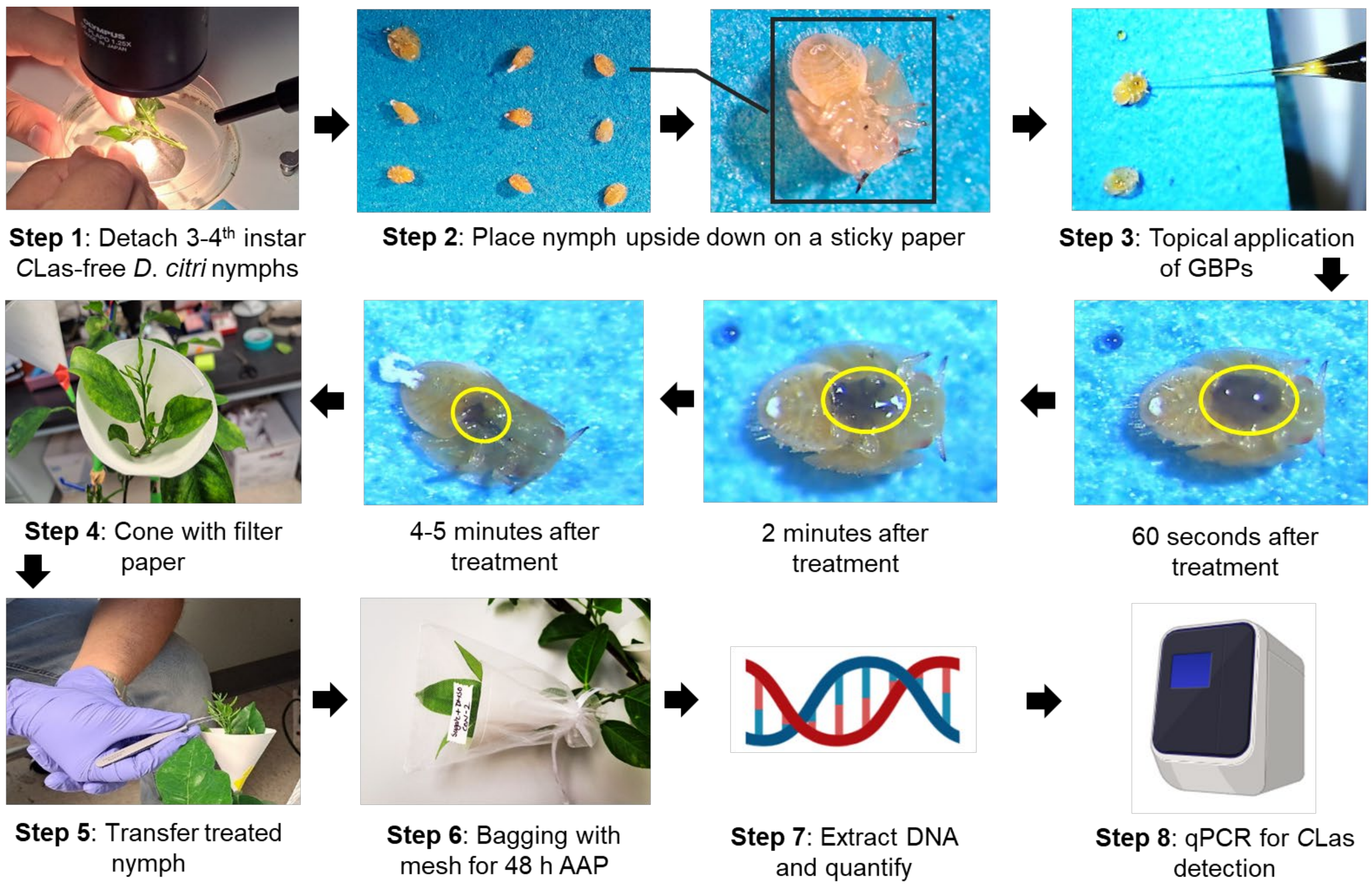

Supplementary Figure S5: Experimental workflow for immobilized droplet feeding assay (IDFA) and *Candidatus* Liberibacter asiaticus (*C*Las) acquisition assay in *Diaphorina citri* nymphs. Third-to-fourth instar nymphs were used for the acquisition assay. The IDFA assay includes the preparation of nymphs on sticky paper, GBP treatment application, cone preparation, transferring nymph to *C*Las-positive plants, DNA extraction, and qPCR analysis for *C*Las detection and quantification.

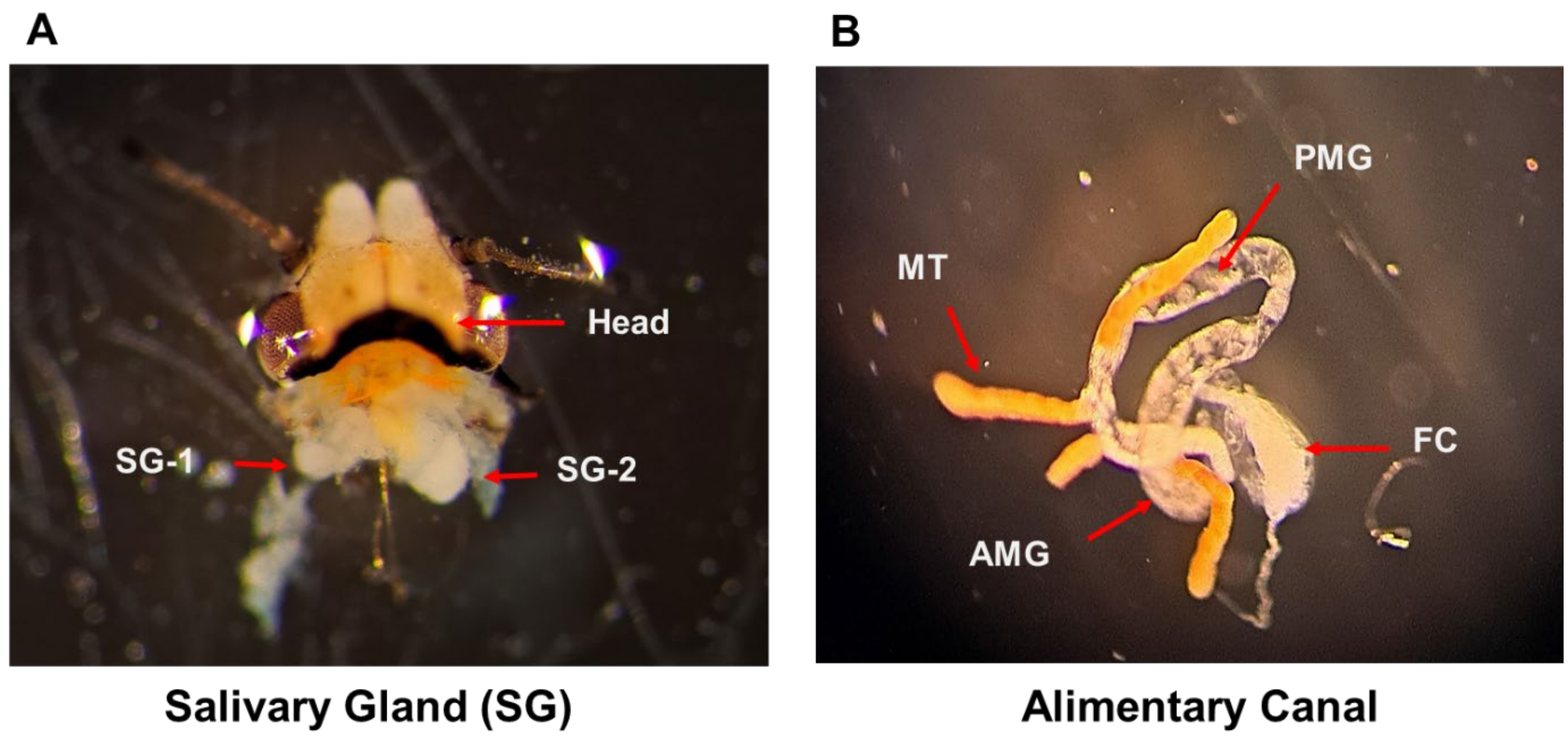

Supplementary Figure S6: Dissected *Diaphorina citri* adult A) salivary glands and B) alimentary canal. SG, salivary gland; MT, Malpighian tubules; AMG, anterior midgut; PMG, posterior midgut; FC, filter chamber.

Supplementary Information

Supplementary Table 1. List of primers and probes used to detect *Diaphorina citri* housekeeping gene (*wingless*), *Candidatus* Liberibacter asiaticus (*C*Las) *16S rDNA*, and host plant (*Citrus sinensis*) internal control (cytochrome oxidase, COX) using quantitative PCR.

Target species	Target gene	Primer/ probe sequence	References
*C*Las	*16S rDNA*	P -50-AGACGGGTGAGTAACGCG-30	[2] Li et al., 2006
		F-50-GCGTTATCCCGTAGAAAAAGGTAG-30	
		R -50-TCGAGCGCGTATGCGAATAC-30	
C. sinensis	*cytochrome oxidase*	P -50-ATCCAGATGCTTACGCTGG-30	
		F-50-GTATGCCACGTCGCATTCCAGA-30	
		R -50-GAATGCCCTTAGCAGTTTTGGC-30	
D. citri	*wingless*	P -50-GCTCTCAAAGATCGGTTTGACGG-30	[3] Li et al., 2008
		F-50-GCTGCCACGAACGTTACCTTC-30	
		R-50-TTACTGACCATCACTCTGGACGC-30	

References cited:

2. Li W, Hartung JS, Levy L, Quantitative real-time PCR for detection and identification of Candidatus Liberibacter species associated with citrus huanglongbing. *J Microbiol Methods* **66**(1):104-15 (2006).
3. Li W DY, Brlansky RH, Twieg E, Levy L, Incidence and population of 'Candidatus Liberibacter asiaticus' in Asian citrus psyllids (Diaphorina citri) on citrus plants affected by Huanglongbing in Florida. *In Proceedings of the 2008 International Research Conference Huanglongbing Orlando, FL* (2008).
